# Predictive Spatial Dynamics and Strategic Planning for Raccoon Rabies Emergence in Ohio

**DOI:** 10.1371/journal.pbio.0030088

**Published:** 2005-03-01

**Authors:** Colin A Russell, David L Smith, James E Childs, Leslie A Real

**Affiliations:** **1**Department of ZoologyUniversity of CambridgeUnited Kingdom; **2**Fogarty International CenterBethesda, Maryland; **3**Department of Biology and Center for Disease Ecology, Emory UniversityAtlanta, GeorgiaUnited States of America; Princeton UniversityUnited States of America

## Abstract

Rabies is an important public health concern in North America because of recent epidemics of a rabies virus variant associated with raccoons. The costs associated with surveillance, diagnostic testing, and post-exposure treatment of humans exposed to rabies have fostered coordinated efforts to control rabies spread by distributing an oral rabies vaccine to wild raccoons. Authorities have tried to contain westward expansion of the epidemic front of raccoon-associated rabies via a vaccine corridor established in counties of eastern Ohio, western Pennsylvania, and West Virginia. Although sporadic cases of rabies have been identified in Ohio since oral rabies vaccine distribution in 1998, the first evidence of a significant breach in this vaccine corridor was not detected until 2004 in Lake County, Ohio. Herein, we forecast the spatial spread of rabies in Ohio from this breach using a stochastic spatial model that was first developed for exploratory data analysis in Connecticut and next used to successfully hind-cast wave-front dynamics of rabies spread across New York. The projections, based on expansion from the Lake County breach, are strongly affected by the spread of rabies by rare, but unpredictable long-distance translocation of rabid raccoons; rabies may traverse central Ohio at a rate 2.5-fold greater than previously analyzed wildlife epidemics. Using prior estimates of the impact of local heterogeneities on wave-front propagation and of the time lag between surveillance-based detection of an initial rabies case to full-blown epidemic, specific regions within the state are identified for vaccine delivery and expanded surveillance effort.

## Abstract 2

A model predicting that the spread of rabies across Ohio will be much more rapid than elsewhere reveals the power of this approach to pro-actively assist targeted surveillance strategies and vaccine delivery

## Introduction

Major recommendations from several Centers for Disease Control and Prevention (CDC) and Institute of Medicine reports on emerging diseases underscore the importance of improving and developing new surveillance strategies to better inform interventions limiting the impact of novel and reemerging disease [1,2,3,4]. By using predictive models of spread we can position ourselves to target surveillance activities and prepare emergency response interventions tailored to the particular feature of an outbreak.

Rabies emergence in the eastern United States and Canada provides an excellent example of how predictive models can help guide surveillance and intervention strategies. Though long endemic in the southeastern U.S., raccoon rabies expanded rapidly along the eastern seaboard during the 1980s and 1990s from an initial focus along the West Virginia–Virginia border; the initial focus was linked to the long-distance translocation (LDT) of rabid animals from Florida [5]. The particular variant of rabies virus associated with raccoons [6,7,8] has spread as a heterogeneous wave away from its original site of introduction and now extends as far north as Ontario [9] and as far west as eastern Ohio, Tennessee, and Alabama [10]. Understanding the context in which models of rabies spread were first developed requires a brief background explanation of the history of epidemic raccoon rabies and the methods used to control spread.

Since late 1997–early 1998, the westward expansion of rabies in raccoons has been curtailed through a massive program of oral rabies vaccine (ORV) delivery focused in eastern Ohio, and later expanded into adjacent border counties in Pennsylvania and West Virginia. Organized by the Ohio Departments of Health, Natural Resources, and Agriculture, in collaboration with the U.S. Department of Agriculture, the CDC, and Canadian agencies, a vaccine *cordon sanitaire* was constructed to keep raccoon rabies out of Ohio [11,12]; it also served the greater purpose of potentially preventing the raccoon variant's spread throughout the geographic range of raccoons, which, with minor exceptions in the southwest, includes the entire continental U.S. south of Alaska [13]. The campaign was successful, and through 2003, raccoon rabies in Ohio was limited to a few sporadic cases within the vaccine zone [10]. In 2004, this comforting streak of success came to an abrupt end.

On 21 July 2004, approximately 11 km beyond the western extent of the vaccine corridor, a rabid raccoon was detected in Leroy Township, Lake County, Ohio [14] (Figure 1). As of 12 August, at least ten additional rabid raccoons had been detected in Lake County and surrounding counties (Figure 1); given the delay of 3–4 mo between first detection of rabies and development of a full-blown epidemic [15,16,17], and the 40-km linear extent of this outbreak, we assume this is a new rabies focus with the potential to engender continued spread of raccoon rabies into Ohio and areas to the west.

**Figure 1:**
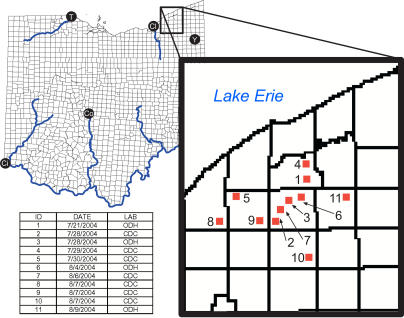
Spatial Location of All Positive Raccoon Rabies Cases in Eastern Ohio as of 11 August 2004 Each sample is identified by number, date of collection, and the laboratory responsible for the positive identification (ODH indicates Ohio Department of Health). To date, more than 300 raccoons have been submitted to the CDC for testing. Ci, Cincinnati; Cl, Cleveland; Co, Columbus; T, Toledo; Y, Youngstown.

Various state agencies have already initiated a remedial wildlife vaccination program to limit further expansion of this epidemic. However, for strategic intervention purposes, determining the expected trajectory and velocity of rabies spread and estimating an effective zone for remedial vaccination would be invaluable [18]. In this paper, we use previously verified mathematical models for predicting rabies spatial dynamics to provide guidance on the likely trajectory and velocity of rabies spread from this emerging focus of infection and to identify specific areas for vaccine delivery and active surveillance.

## Results/Discussion

### The Raccoon Rabies Epidemic

When the expanding wave front of raccoon rabies reached the borders of Connecticut (CT) and New York (NY), county-level counts of animal rabies data were already being reported monthly to the CDC, as required for this nationally notifiable disease. Previously we analyzed time series data from the county-level data to make predictions about the temporal structure of recurring epidemics and estimated the lag time (approximately 3–4 mo) from detection of raccoon rabies to epizootic development [15,16]. In separate analyses, we arrived at similar estimates for surveillance delays [17]. The county-level data were suitable for assessing a general rate of front movement across the northeastern U.S. but not sufficiently resolved so as to provide information about local environmental heterogeneities that may alter rates of spread.

Concurrent with the collection of county-level data, animal rabies cases occurring within individual townships were also collected by NY and CT state health departments, increasing the spatial resolution of disease reporting data 20-fold above that available from county-level reports. This increase in spatial resolution allowed for the construction of a detailed spatial model of rabies spread.

### The Model

The data from NY and CT have been used to parameterize a stochastic spatial model for rabies spread among townships (Figure 2). The model was used for an exploratory data analysis to quantify the spatial dynamics across CT, and we found a 7-fold reduction in local transmission when geographic regions (i.e., townships) were separated by major rivers. In addition, the LDT of rabid animals was incorporated into the model to accommodate these rare, but significant events, often capable of engendering epizootics well in advance of the wave front when larger spatial domains were examined. The spatial model parameterized for CT was used to hind-cast the time to first appearance of raccoon rabies across NY townships using only knowledge of where the disease was introduced into the state and local heterogeneities that may influence the rates of spread [19]. The a priori CT model was able to predict the pattern of spread across NY, as well as provide an estimate of the possible effect of ORV intervention. Having demonstrated the predictive power of the spatial stochastic simulator for CT and NY, we can now use the model and our knowledge of the current outbreak to strategically forecast the spatial spread of rabies in Ohio.

**Figure 2:**
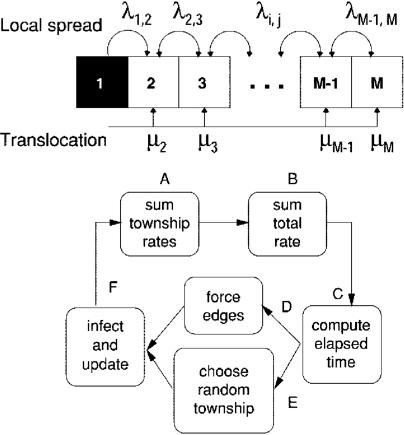
A Stochastic Model Was Used to Simulate the Heterogeneous Spread of Raccoon Rabies on an Irregular Network, Illustrated Here on a Simple Array An infected township *i* infects its adjacent neighbor *j* at rate λ*_ij_*. In addition, township *j* may become infected because of translocation of rabid raccoons at rate μ*_j_*. Heterogeneity was incorporated by allowing the local rate of infection from neighboring townships, and the rate of translocation, μ*_j_,* to be different in different models. Each algorithm for associating a set of rates with rivers defines a stochastic candidate model. The simulation algorithm involved six steps. (A) For each township, add the rates of infection from all possible routes of infection. (B) Add the townships rates to compute a total rate. (C) Draw a random number to determine the elapsed time. (D) Check to see if any forced townships have become infected in the elapsed interval. (E) If no edges were forced, select a random township to infect. (F) Infect the forced township, update the local rates, and repeat until each township becomes infected (after [17]).

The model transforms Ohio townships into a network; local spread of raccoon rabies among adjacent townships in the Ohio model was predicted where two townships shared at least one common point along their borders. An infected township *i* was assumed to infect its adjacent neighbor *j* at rate λ*_ij_*. In addition, long-distance dispersal of rabies was incorporated by assuming a low and constant rate of global infection μ*_j_* for all uninfected townships regardless of spatial proximity to infected neighbors (Figure 2).

Incorporating environmental heterogeneities into local rates of spread is crucial to predicting the local wave-front movement of terrestrial rabies. Rabies transmission was 7-fold lower when townships were separated by a river in CT, while in NY, the Adirondack mountain range was, and remains, an impenetrable barrier to the incursion of raccoon rabies. Ohio lacks the rough mountainous terrain of NY, but it has several major rivers that could influence westward expansion of raccoon rabies from a focus near the Pennsylvania or West Virginia border; five major Ohio rivers, the Miami, Muskingum, Scioto, Maumee, and Cuyahoga Rivers, were incorporated into our simulations (Figure 3).

**Figure 3:**
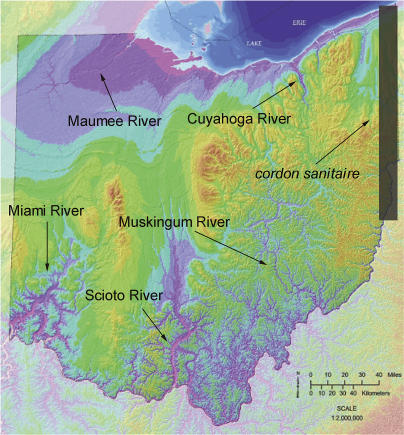
Satellite Image of Ohio Topography Illustrating Major River Systems and Location of Vaccine Corridor Extent and shape of the vaccine corridor is approximated. (Map reproduced with permission from Ohio Department of Natural Resources, Division of Geological Survey.)

### The Ohio Forecast

Transposing the model for use in Ohio merits a discussion of the geographic differences between CT, NY, and Ohio. In CT, the Connecticut River bisects the state, running north to south. The river's effect on the epidemic was maximized because the direction of epidemic spread ran orthogonal to the river, i.e., from west to east. Moreover, CT is much smaller than NY or Ohio. When the model was transposed into NY from CT, the townships in the Adirondack Mountains were excluded a priori because none of them had ever reported a case of raccoon rabies. The general triangular shape of NY and the exclusion of the Adirondack townships from the simulation, in conjunction with there being three initial foci within NY, left few townships far from any single initial focus. Thus, the successful hind-cast was based mostly on the relatively predictable local dynamics. In contrast, in Ohio we know of only one focus, in the northeastern quarter of the state, leaving many townships hundreds of kilometers from the epidemic focus. Given the greater distance between the epidemic focus and far reaching townships, LDT of infected individuals in Ohio could have a much more profound impact on the wave-front dynamics than was possible in NY or CT. Given the unpredictable nature of LDT events, we present two projections. In the first projection, we ignored LDT and focused on local rabies wave-front movement: λ*_ij_* = α = 0.66 for townships not separated by a river; λ*_ij_* = β = 0.12 for townships separated by a river; and μ = 0. In the second projection, we used the exact parameters fitted to the CT epidemic: λ*_ij_* = α = 0.66 for townships not separated by a river; λ*_ij_* = β = 0.12 for townships separated by a river; and μ = 0.0002.

The local-spread-only projection for Ohio assumed the rabies wave-front origin was from the townships nearest the center of the Lake County outbreak (case 3 in Figure 1), and the epidemic was simulated without LDT. As the local dynamics of rabies are fairly predictable, this could be considered the best-case scenario. Retaining the parameters for local spread from CT and disallowing any LDT, the rabies epidemic required 70 mo to reach the western corners of the state (Figure 4A). In the absence of LDT, the predicted rate of front propagation is faster than previously reported for other states because of the influence of the permissive zone of central Ohio, in which few environmental impediments exist.

**Figure 4:**
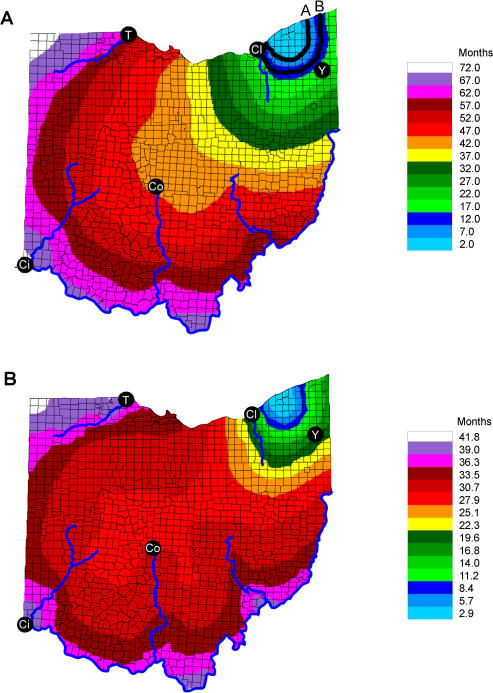
Predicted Trajectory of Rabies in Ohio Based on the Current Outbreak (A) Predicted time to first appearance (months) of raccoon rabies spreading from Chardon Township and surrounding townships given no vaccine intervention and with LDT excluded from model predictions. The time course of spread is revealed through the contour plot, where each color band indicates a given time interval to arrival at a township. The width of the bands corresponds to velocity of spread, with wider bands associated with more rapid spread. Major cities are Cleveland (Cl), Youngstown (Y), Toledo (T), Columbus (Co), and Cincinnati (Ci). The two black lines labeled A and B correspond to the area where cases have been detected (A) and the expected position of the wave front given a long-tailed distribution of incubation periods. (B) Predicted time to first appearance (months) of raccoon rabies spreading from Chardon Township and surrounding townships given no vaccine intervention and including estimates of long-distance dispersal modeled in CT and NY .

The projection with LDT was approximately one-third faster than the local-only spread, even with the very low levels of long-distance dispersal modeled. In these simulations rabies spread across central Ohio within 33 mo and covered the state by month 41 (Figure 4B). Passage across the midsection of Ohio was particularly fast; the estimate of 100 km/y far exceeds previous estimates for the rate of spread of raccoon rabies, which typically ranges between 30 and 60 km/y [20,21,22]. By our estimates, if unchecked, rabies will likely spread across Ohio in the same amount of time that it took rabies to transverse CT, even though Ohio is 2.1 times wider than CT. The potential for such rapid spread is quite alarming.

Adding the possibility of global LDT to the model, even at the low rate assumed in the original model for CT, served as a massive promoter of rapid spread. This can be envisaged as the worst-case scenario, with LDT greatly increasing the rate of wave-front propagation across the state. Because of the largely unpredictable nature of LDT, it must be assumed that reality is likely to be somewhere in between the two modeled scenarios, but exactly where is difficult to discern.

The picture that emerges from both scenarios suggests that rabies will spread rapidly through the middle of Ohio, where there are few environmental impediments to disease front expansion. Rivers and mountains in Ohio do not constitute a major barrier to rabies spread. Similarly, rabies will not encounter any impediment like the Adirondack Mountains in NY. With no effective physical barrier across the middle of Ohio, rabies could move more rapidly through this zone then in any previously recorded epizootic.

The design of vaccine barriers to control the Lake County epidemic must allow for the distance the wave front will advance prior to detection of the first laboratory-confirmed case of raccoon variant rabies within a county. This parameter is unknown, although estimates of the delay from the first detected case of raccoon variant rabies to the start of the first epidemic exist [15,16,17,18]: the delay is approximately 3–4 mo. Thus, the detection of the first case of raccoon rabies in a township through passive surveillance indicates roughly where the front was 3–4 mo before.

As no other data exist to help assess the bias in lag between the arrival date of raccoon rabies and the date of detection recorded in the national surveillance database, we set the interval from arrival to detection at 3–4 mo. This parameter defines where to place the inner perimeter line A (Figure 4A). Few estimates of incubation time from natural field situations exist; however, Tinline et al. [18] provided some estimates on the distribution of incubation times for raccoons infected with their homologous variant. We used the 75th percentile in incubation time from Tinline et al. [18] to derive a confidence line for the expected wave-front advance (line B in Figure 4A), adding the incubation time to the calculations for line A; thus the area between perimeter lines A and B defines the vaccination corridor required to target susceptible animals (Figure 4A).

### Strategic Planning and Alternative Outbreak Scenarios

Any intervention decisions or strategic plans based on model projections require that models deliver a robust outcome. We explored two additional scenarios for raccoon rabies emergence and spread through Ohio based on potential breach points in the vaccine corridor and consider the utility of the model for selecting sights for enhanced surveillance.

#### Alternative scenario 1

A breech in the *cordon sanitaire* could occur within a similar time frame as a LDT, and we considered the effects of independent and concomitant breeching and LDT events in a series of alternative scenarios. Adding such events to the spatial simulations significantly decreases the times to first appearance of raccoon rabies compared to that predicted solely on local spread. Modeling various LDT scenarios is warranted because LDTs occur frequently [23] and have demonstrably played a central role in the epizootiology of raccoon rabies, and are regarded herein and elsewhere as a critical element in our efforts to understand and model the epizootic dynamics of raccoon rabies.

We simulated the pattern of rabies spread associated with a breach in the vaccine corridor near Youngstown (near the current outbreak location) coupled with a forced LDT event into the Athens-Hocking landfill. The Athens-Hocking landfill is the landfill closest to the current front of raccoon rabies outside of Ohio that receives interstate refuse. The LDT event was incorporated into the computer simulation as an initial condition that occurred at month 12 during the simulated spread, in addition to base levels of LDT derived from CT (μ = 0.0002). Spread from the landfill in conjunction with the advancing front originating near Youngstown generated two advancing fronts that converged in the middle of the state (Figure 5A).

**Figure 5:**
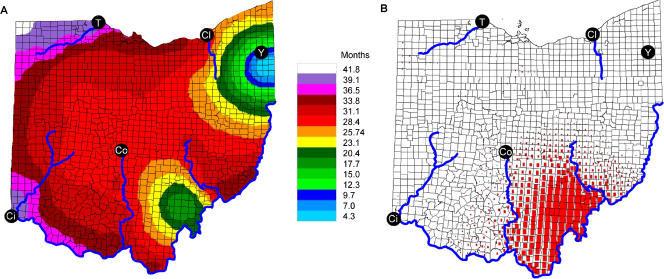
Combined Effects of LDT and a Break in the Vaccine Barrier on Pattern of Rabies Spread across Ohio (A) Predicted time to first appearance (months) of raccoon rabies given a breach of the *cordon sanitaire* near Youngstown and the LDT of an infected individual into the Athens-Hocking landfill at month 12. (B) Residual differences between simulations of the Youngstown breach with and without the forced LDT event at the Athens-Hocking landfill. The larger the red squares, the larger the residual difference between the simulations with and without the forced LDT. Ci, Cincinnati; Cl, Cleveland; Co, Columbus; T, Toledo; Y, Youngstown.

To explore the overall impact of multiple introductions on the time to first appearance we compared simulations with and without the translocation event into the Athens-Hocking landfill. The forced LDT event caused rabies to reach the southern portion of Ohio far earlier than predicted in the absence of the forced LDT. However, the rivers seem to limit the overall additive effect on time of first appearance in western Ohio by acting as natural barriers to spread. There appears to be no additive effect on the rate of disease movement across the middle of the state associated with the union of the advancing wave fronts, as revealed in a plot of the residual differences between simulations (Figure 5B).

#### Alternative scenario 2

Rabies has a variety of alternative paths of entry into Ohio from infected regions across the *cordon sanitaire*. In this scenario, we consider the spatial trajectories of rabies spread from a variety of single points of entry and compare these alternative trajectories to determine the most common areas of overlap and, hence, where rabies is most likely to emerge. For example, we consider the trajectory of spread given an introduction around Youngstown and compare this with an introduction around Bridgeport, Ohio (near Wheeling, West Virginia), that would be near the southern end of the vaccine corridor in Ohio.

It is reasonable to assume that the Ohio River serves as a barrier to raccoon movement, but there is the potential for rabid individuals to cross bridges into Ohio. Given an introduction at Bridgeport, Ohio (Figure 6), the model predicts that the initial westward expansion would be halted by the Muskingum River, while northward expansion would be rapid. Following a crossing of the Muskingum, the model predicts the same sort of rapid westward expansion seen in the first scenario.

**Figure 6:**
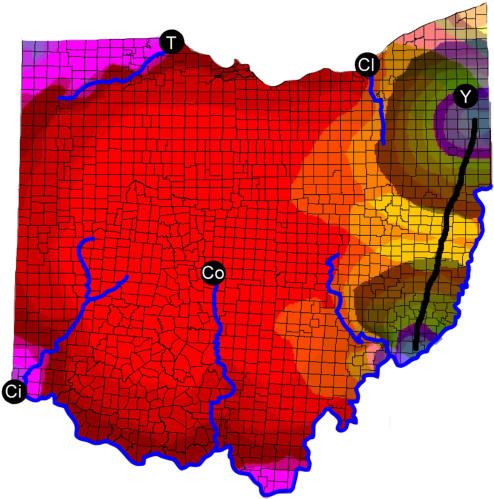
Combination of Predicted Time to First Appearance Projections from Youngstown Breach and Ohio River Breach Scenarios Thick black line indicates the minimal distance between the two epidemic foci orthogonal to the temporal contours. Given the trajectories, the path corresponds to the most likely location where the waves will first collide.

#### Scenario comparison

By jointly considering the predictions from these introduction scenarios, we demonstrate the utility of our model in establishing sentinel points for surveillance. Figure 6 shows the model predictions for spread associated with a river crossing near the southern terminus of the *cordon sanitaire* overlaid with our projections for spread away from the Youngstown area introduction. From the combination of these two scenarios we see that the first point at which the two epidemic fronts are predicted to meet is not along the Ohio–West Virginia border but further west towards the interior of the state. We can use this juxtaposition of the two trajectories to establish a minimum time path for the epidemic movement (Figure 6) and can suggest sentinel points for surveillance along this line in addition to the surveillance strategies already in place.

The three simulation sets—the current outbreak (with LDT) and alternative scenarios 1 and 2—can be compared to assess the sensitivity of rate estimation on at least these initial conditions. A plot of the maximum residual differences in time to first appearance between all three simulation sets suggests that the predicted velocity and overall trajectory of rabies spread are robust to changes in outbreak origin (Figure 7). Though large residuals exist between the three simulations around the introduction sites, a striking common feature of all simulation outcomes is the rapid spread of raccoon rabies across central Ohio given an average effect of LDT. Maximum differences in the estimated time to raccoon rabies arrival between simulations in central and western Ohio varied by less than 1 mo, irrespective of breach location in the ORV barrier.

**Figure 7:**
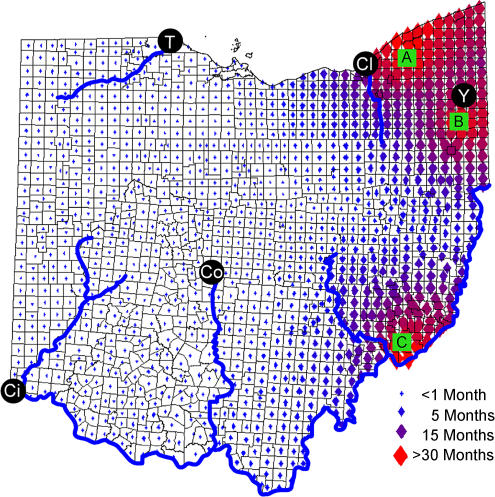
Residual Differences between Time to First Appearance from Simulated Epidemics Initiated by Breaches in Different Geographic Locations The maximum difference between the predicted times to first appearance is indicated in each township by the size and color of the corresponding diamond. (A) represents an epidemic simulated from current outbreak data shown in Figure 1. (B) represents an epidemic simulated from the movement of an infected animal across the *cordon sanitaire* near Youngstown. (C) represents the introduction of an infected animal from West Virginia at a bridge across the Ohio River.

### Conclusions

Experience with parenteral rabies vaccination programs and ORV-based attempts to control wildlife rabies in the U.S. and Europe has demonstrated that all vaccine barriers are, to some extent, permeable and vulnerable to breaches [24,25,26]. Ohio has proven to be no exception.

Considering the vulnerabilities of any vaccine corridor, and the difficulties inherent in anticipating break sites in a vaccine barrier and identifying sites of LDT, the unlimited ability to explore scenarios by mathematical simulation and to compute and compare different design strategies for ORV interventions provide a valuable exercise to plan for real epidemics. From the simulations shown here, a robust pattern of trajectory, an extraordinarily high rate of spread of raccoon rabies through central Ohio, the significant potential impact of LDT, and lack of environmental impediments suggest that a strategy combining early detection and rapid intervention are requisite if control is to succeed. A robust intervention plan is invaluable, even if the exact site or sites to which it is applied prove elusive*.*

The approach discussed here reveals some of the many gaps in our knowledge of wildlife rabies and in the data available to inform initial conditions for simulations. Without a second measure of the arrival–detection delay, obtained from an independent and extremely sensitive surveillance system (e.g., coupling active sampling of road-killed raccoons, purposeful hunter collections, and kill-trap data), we have no tools to calibrate detection times obtained through the passive national surveillance system.

In western Europe, reinforcement of natural barriers, such as rivers, lakes, and mountains, have been a staple in the successful campaign to control red fox rabies by vaccination [24]. In Massachusetts and NY, pre-existing natural (i.e., the Adirondacks) and artificial (i.e., the Cape Cod canal [27]) impediments to the free movement of raccoons, and, hence, raccoon rabies, were reinforced by vaccine distributions. Unfortunately for the remedial efforts to contain the Lake County focus in Ohio, the benefits derived from enhancement of natural barriers are not an option. With the exception of river systems in southeastern Ohio, such as the Muskingum (for topographic details see Figure 3), there are few natural barriers to augment ORV distribution in the Lake County region or in central Ohio, if required.

As reinforced natural barriers are not an option for rabies control in much of Ohio, the need for rapid remedial intervention by ORV and intensified, active surveillance to estimate the actual epidemic boundaries is immediate. Enhanced surveillance, perhaps by collection and rabies testing of road-killed raccoons, and stockpiling of ORV sufficient to establish a new ORV barrier in central Ohio, are prudent courses of action.

If the Lake County epidemic escapes beyond remedial intervention efforts, the fallback position demands rapid construction of a second *cordon sanitaire* spanning central Ohio, at a location dictated by the then current position of the epidemic. If the disease is not confined within Ohio, the limits to raccoon variant rabies spread are defined only by the geographic distribution of its host. Nothing short of these activities will contain a rapidly expanding and fast-moving epidemic of raccoon rabies, should ring vaccination and other regional efforts fail to eliminate the current focus around Lake County. The interventions described here would appear as inexpensive alternatives to the uncontrolled westward spread of rabies and the loss of the millions of dollars invested in what would become the vaccine equivalent of the Maginot Line.

## Abbreviations

CDC: Centers for Disease Control and Prevention
CT: Connecticut
LDT: long-distance translocation
NY: New York
ORV: oral rabies vaccine
